# Principal Component Analysis of Gait Kinematics Data in Acute and Chronic Stroke Patients

**DOI:** 10.1155/2012/649743

**Published:** 2012-02-15

**Authors:** Ivana Milovanović, Dejan B. Popović

**Affiliations:** ^1^Faculty of Electrical Engineering, University of Belgrade, 11120 Belgrade, Serbia; ^2^Tecnalia Serbia D.O.O., 11000 Belgrade, Serbia; ^3^Center for Sensory Motor Interaction (SMI), Aalborg University, 9220 Aalborg, Denmark

## Abstract

We present the joint angles analysis by means of the principal component analysis (PCA). The data from twenty-seven acute and chronic hemiplegic patients were used and compared with data from five healthy subjects. The data were collected during walking along a 10-meter long path. The PCA was applied on a data set consisting of hip, knee, and ankle joint angles of the paretic and the nonparetic leg. The results point to significant differences in joint synergies between the acute and chronic hemiplegic patients that are not revealed when applying typical methods for gait assessment (clinical scores, gait speed, and gait symmetry). The results suggest that the PCA allows classification of the origin for the deficit in the gait when compared to healthy subjects; hence, the most appropriate treatment can be applied in the rehabilitation.

## 1. Introduction

Neurological deficits caused by stroke lead among other things to loss of leg strength, impaired balance, spasticity, and rigidity, all affecting the ability to walk [1]. The inability to walk directly leads to so-called no-use pattern, and this diminishes cardiovascular fitness and contributes further to disability. Thereby, it is important to restore walking to the level that allows social activities. Currently, body-weight supported gait on a treadmill and use of various types of assistive systems to induce walking alike activity suggest benefits. The meta-analysis, however, shows limited improvement compared to traditional treatments [2–6].

This study is part of the clinical study of the efficacy of the device Walkaround for assistance to posture while exercising walking. The Walkaround provides partial body-weight support, forces the progression at the preset speed (0.1–1.2 m/s), eliminates the need for hand support, prevents falls during gait training sessions, and provides longer training [7]. The Walkaround allows free or assisted leg joints rotations. The joint movements can be additionally assisted with both external skeleton or functional electrical stimulation systems.

The outcome measures of this clinical study include kinematical measures (gait speed, symmetry of gait, cadence and other temporal parameters, ground reaction forces, and joint angles), and clinical measures (Functional Ambulation Categories: 0 to 5 [8], Fugl-Meyer score for lower extremities: 0–36 [9], and Berg balance test: 0–56 [10]). The analysis of data at the beginning of this study indicated significant differences in measures of the gait that are not typically included in the reports of clinical studies. Namely, the clinical measures were very similar between the groups, but the differences in kinematic synergies were significant. This implicates that one needs to consider kinematics in details when selecting the most appropriate therapy.

## 2. Methods

### 2.1. Subjects

Thirty-two subjects were recruited for the randomized study. Twenty-seven patients were selected based on the inclusion criteria: first ever stroke, acute or chronic stage of hemiplegia; unilateral weakness; ability to walk at least 10 meters with or without an assistive device or hand support; cognitive ability to follow the instructions. 16 subjects were in the chronic group (CG) (more than 6 months after stroke), and 11 subjects in the acute and subacute group (AG). The AG was composed of four patients in acute stage (less than 6 weeks after stroke) and seven patients in the subacute stage (6 weeks to 6 months after first stroke). All subjects signed an informed consent form, approved by the local ethics committee. Subject characteristics are summarized in Table 1.

The Fugl-Meyer for lower extremities (min 0, max 36), Berg Balance test (min 0, max 56) and Functional Ambulation Category (min 0, max 5) scoring was performed by a single certified experienced physical therapist in all patients, to eliminate the interrater variability.

In addition, the data when walking at slow pace (*v* = 0.4 to 0.6 m/s) from five healthy subjects (age 62 ± 3) with no known neurological or orthopedic problem were used as the benchmark. Healthy subjects signed the informed consent approved by the local ethics committee before the measurements.

### 2.2. Instrumentation

**Figure 1:**
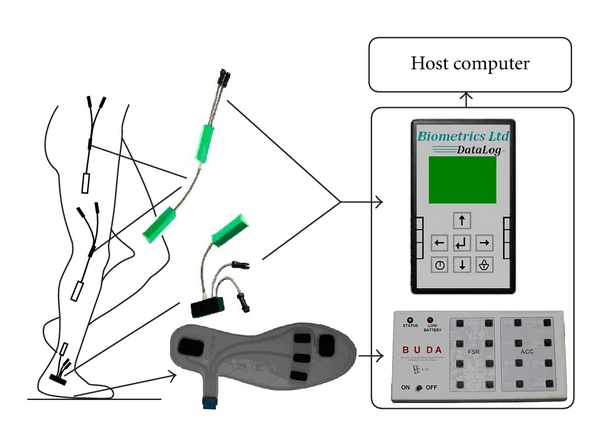
The data acquisition instrument with sensors used for the recordings of ground reaction forces and gait kinematics. Biometrics DataLog system was used for signal conditioning coming from the flexible goniometers. The BUDA is the microcomputer acquisition module for processing and wireless communication with the remote computer [11].

The kinematics of gait was acquired with a portable multisensor system for gait analysis (Figure 1). The core of the instrument is a data acquisition portable device which transmits signals to a remote PC via Bluetooth [11, 12]. The set of sensors used in this study comprised six goniometers (SG110 for ankle joint, SG150 for knee and hip joints; Biometrics Ltd., Gwent, UK) and two shoe insoles instrumented with five force sensing resistors each [11] for assessing ground reaction forces. Goniometers were mounted using double-sided adhesive tape and positioned across hip, knee, and ankle joints on both legs following the instructions of the manufacturer. Sensor insoles were placed in both, left and right shoe, measuring ground reaction forces under toe, metatarsal, and heel zone (Figure 1). Signals were recorded at a sampling rate of 166 Hz.

### 2.3. Protocol

Subjects were asked to walk along a 10-meter long straight-line path, at a self-selected, comfortable speed. Ground reaction forces and joint angles data were recorded during the gait. At least 12 gait trials were recorded for each subject in three consecutive days. Patients were allowed to rest for at least five minutes between the gait trials on the same day to minimize effects of fatigue. The sessions were also video-taped for later analysis of unexpected events in the kinematic and dynamic data.

### 2.4. Data Processing

The data was truncated to individual steps. The extraction of individual steps was done automatically by the threshold method. The threshold level was set at 5% of the maximum ground reaction force (heel-contact and toes-off events), and the procedure was used for both the paretic and the nonparetic leg. The steps were then normalized to the 100% in order to allow a detailed analysis. This normalization included fitting of the 100 points in the data determined as one step. Steps that differed for more than 10% of the average step duration in each gait trial were disregarded (less than 4% of all strides). The signals were initially low-pass filtered using a second-order Butterworth filter (*f*_c_ = 5 Hz) and further processed, and then filtered at 30 Hz with the fourth-order zero phase shift Butterworth filter.

The novelty in this data processing is the application of the Principal Component Analysis (PCA) to each of several data sets consisting of normalized gait patterns over a step cycle. The PCA is a way of identifying patterns in data and expressing the data in such a way as to highlight their similarities and differences. Since patterns in data can be hard to find in data of high dimension, where the luxury of graphical representation is not available, PCA is a powerful tool for analyzing data. The other main advantage of PCA is that one founds these patterns in the data, and compresses the data, reduces the number of dimensions, with minimum loss of information. We selected this method based on important findings related to the characterization of gait [13, 14]. In factorial analysis, the basic waveforms are determined by the structure of the data waveforms. The analysis involves calculation of the correlation matrix, extraction of the initial principal components, application of the varimax rotation, calculation of factor scores, and interpretation of the results. The principal components (PCs) were expressed using a varimax rotation in order to minimize the number of variables with high loadings on each component factor [15]. This is simplifying the interpretation of the PCs since the waveforms of the rotated factors are closer to the original signals [16, 17].

The appropriate application of the analysis involves an initial estimate of the extent to which each data waveform is composed of components common to other data waves, the communality, and the extent to which activity is specific to each wave alone, the uniqueness [18]. We hypothesized that the waveforms in our case are being dependent on two aspects: joint synergies characteristic for cyclic type behavior and unique aspect of motor activity associated with a single, not other joints.

We selected the Bartlett's test of sphericity to assess whether the dataset is adequate for factor analysis. The Bartlett's test of sphericity examines the hypothesis that the correlation matrix comes from a population in which the variables are independent. Rejection of the independence hypothesis is an indication that the data are adequate to factor analysis. Another important element that we included was that eigenvectors with the corresponding eigenvalues less than unity are typically related to noise [17, 19]. Hence, we retained only factors with eigenvalues greater than one. This criterion was proposed by Kaiser [15].

In summary, the PCA can be considered as a method for reducing dimension of the data. When applying the method, a set of input variables is transformed into a set of noncorrelated variables (principal components). These variables are a linear combination of originals and ranked according to the amount of variance that they contain. The PCA provides the mapping of original data into orthogonal space, with the principal axes in directions of maximum variance of the original data. By this transformation, the mapping of vectors *x*  (*x* *ϵ* *R* ^*n*^) into a lower-dimensional vectors *y*  (*y* *ϵ* *R* ^*m*^), with *m* < *n*, is allowed. The data covariance matrix can be estimated by

(1) $$S=\frac{1}{N-1}\overset{N}{\underset{1}{\sum }}\left(x_{k}-\mu \right){\left(y_{k}-\mu \right)}^{T},\;\mu =\frac{1}{N}\overset{N}{\underset{1}{\sum }}x_{k},$$

where *N* is the total number of data. In order to determine the orthogonal basis of mapped feature space, the eigenvalue decomposition is performed. The eigenvalues *γ*_*i*_ are in descending order, and *i* = 1,…, *n*))

(2) $$Su_{i}=\gamma_{i}u_{i},\;\gamma_{1}\geq \cdots \gamma_{j}\cdots \geq \gamma_{n}.$$

Taking into consideration the *m* largest *γ*_*i*_ and their corresponding eigenvectors u_i_, the mapping is given with

(3) $$y_{i}={u_{i}}^{T}\left(x-\mu \right),\;i=1,2\ldots ,m.$$

### 2.5. Statistics

Student's *t*-test was used to analyze the differences in measured values between the groups.

## 3. Results

**Figure 2:**
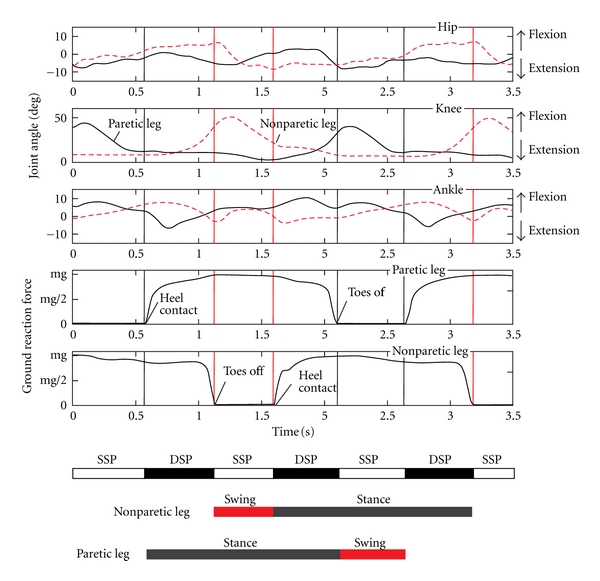
Joint angles and ground reaction forces recorded during one of the gait sessions in one patient. The acronyms SSP and DSP are for single support and double support phases of the gait cycle, and mg is the weight of the subject.

Figure 2 is an example showing a sequence from the preprocessed recordings of joint angles and ground reaction forces (GRFs) of the paretic and the nonparetic leg, with the indications of double support phase (DSP), single support phase (SSP), and swing and stance phases.

**Figure 3:**
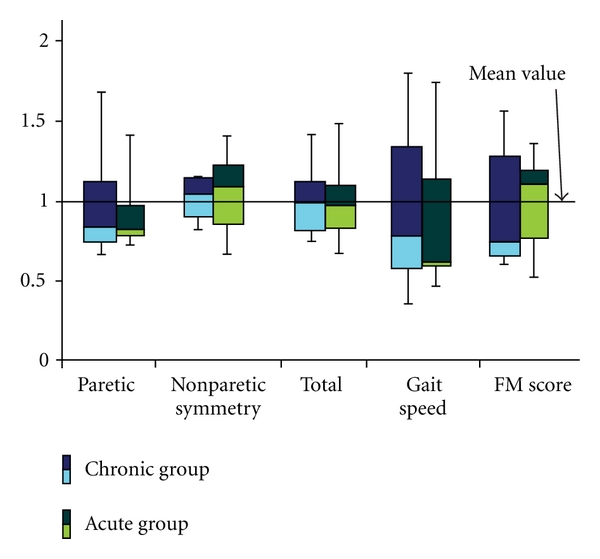
Box plots for the symmetry, gait speed and FM score.

Figure 3 is the box plot with the quartile values for the symmetry index (from paretic leg, to paretic leg and whole stride), gait speed, and FM score normalized to the mean value for each of the groups (CG and AG). There were no significant differences between groups in these variables.

**Figure 4:**
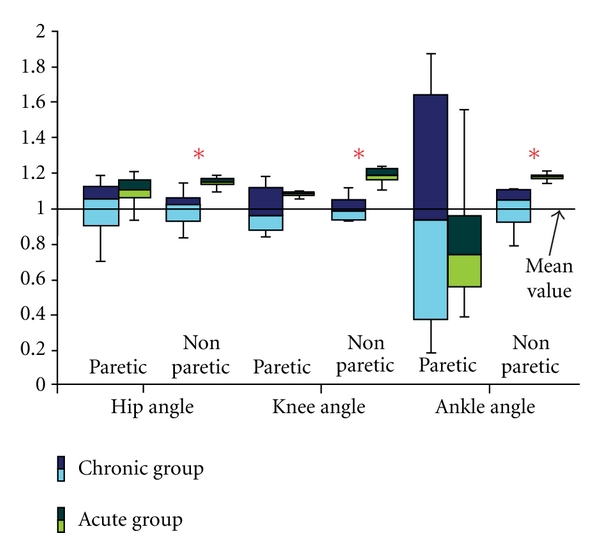
Quartile distribution of the hip, knee, and ankle joints of the paretic and the nonparetic leg in chronic and acute groups. The asterisks denote significant differences (*P* < 0.01).

Figure 4 shows significant differences found between joint angles for the AG and CG. Among all joint angles, significant differences were found only in joint angles of the nonparetic leg. This could be explained by the fact that the dominating intersegmental coordination was lost. This suggestion follows the result of Ivanenko et al. [14] who found that temporal changes of the angles of lower limb joints do not evolve independently, but they are tightly coupled.

### 3.1. Healthy Individuals

**Figure 5:**
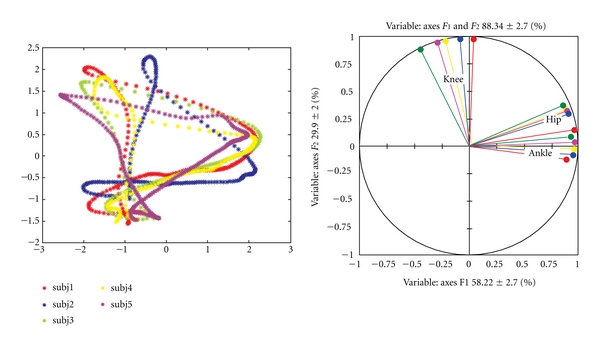
Cyclograms for five healthy subjects forming a characteristic D-like shape (left panel) and Pearson's correlations between the ankle, knee, and hip joint (right panel).

The PCA of joint angles recorded in healthy subjects shows that first two principal components account for about 88% of total variance (from 83% to 94%). First principal component (PC) describes about 58%, while second PC describes about 30% of total variance. Projection of all data points onto the first two components forms a two-dimensional D-like shape (Figure 5). In all data used, the Bartlett's test of sphericity indicated that a PCA was appropriate method (*P* < 0.0001).

The D shapes (Figure 5, left panel), called cyclograms, have been already reported in the literature and used in computer vision applications [20] and gait recognition [21]. The D shapes differ subtly in shape and position among subjects, but always have the same well-recognizable form. Calculations of principal components based on Pearson's correlation matrix gave us an additional insight into mutual dependence of initial variables. Pearson's correlations are presented in Figure 5 (right panel) as vectors of the correlation circle. If vectors are close to each other, they are significantly correlated. If they are orthogonal they are not correlated. We found that, in healthy subjects, hip and knee are negligibly correlated (*r* = 0.09–0.22). Hip and ankle show moderate degree of correlation (*r* = 0.48–0.66), while knee and ankle show variable degree of correlation across subjects, ranging from low to moderate.

**Figure 6:**
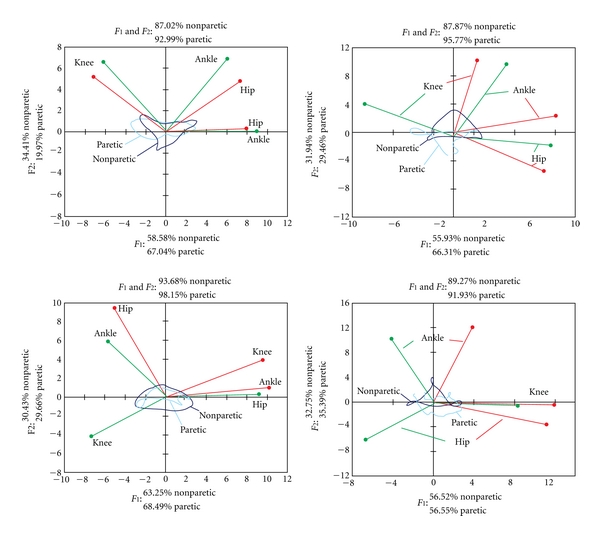
Cyclograms and Pearson correlation coefficients for four stroke patients. The top panels are for subjects who belong to low level of disability, and the bottom for the high level of disability. Left panels show 2 patients in the chronic stage, while the right panels show two patients in the acute stage of hemiplegia.

Figure 6 shows results of the principal component analysis in four stroke patients. We present two patients from the group that had gait speed above *v* = 0.5 m/s and had the FM *≈* 25 (Figure 6, top panels), and two patients that had the gait speed at about *v* = 0.2 m/s and the FM *≈* 20 (Figure 6, bottom panel). The left panels show patients from the CG, and the right panels from AG. The main finding from the analysis is that patients in the acute group have a near-normal cyclogram in the nonparetic leg, suggesting that they have near-healthy leg movement synergy, and that patients in the CG have not only modified synergies in the paretic but also in the nonparetic leg.

The summary of the figures showing the cyclograms is given in Table 2.

## 4. Discussion

The clinical measures (Fugl-Meyer scores, Functional Ambulation Categories, and Berg Balance scores) show no significant differences between chronic and acute stroke patients (Table 1).

The duration of the stride in acute groups was between 1.95 and 3.05 s, while the duration in the chronic group was longer (2.05–3.50 s) yet, not statistically significant. The step lengths were similar in the acute (0.71 and 0.81 m) and chronic (0.66 and 0.78 m) groups. The analysis of other temporal and spatial parameters also did not show significant differences.

A typical measure of the quality of gait is symmetry. Our study indicates that the differences in the symmetry exist between the chronic and acute groups, but these differences were not significant (Figure 3). One of the reasons for this finding was likely a large variation of the symmetry indices in both groups.

The analysis of joint angles of both legs (Figure 4) revealed the differences in kinematics between the AG and CG. The most interesting aspect is the difference that was assessed in the nonparetic leg. Abnormal joint angle patterns found are consistent with clinical characterization of hemiplegic overground gait and are most probably related to hip hike and circumduction.

These differences were further analyzed by means of PCA. We found that in healthy subjects, first two principal components account for majority of joint angle variance. Planar covariance among joint angles, previously reported in [14], represent a constraint that could be explained in terms of mechanics as a reduction of number of degrees of freedom from three to two. Thus, the two joint angles determine the third one.

The analysis of data from patients reveals differences from the healthy gait pattern. As severity of hemiplegia grew, first two PC carried more of the total variance. Also, distribution of variance among components changed. The larger deviation from the normal gait pattern is expressed as an increase of first PC at the expense of the second PC, in some cases, leading to a significant reduction of this component. In terms of joint angles, this provokes stronger linear coupling of three joint angle variables. This linear dependence reduces the possible number of different joint angle combinations.

If we compare healthy gait pattern with planar covariance to more constrained gait leading to almost linear covariance, we can conclude that, in case of injury, neural system loses some of its control possibilities. Ivanenko et al. [14] showed that in case of a constrained gait (in-place movements and overground marching), trajectory collapses to a line and there is linear covariance between angles. So, we could say that as we get closer to linearity (first PC more dominant), gait becomes more constrained.

After analyzing data from acute and chronic hemiplegic patients, we were able to identify some patterns of disturbed behavior. Results are shown in Table 2. We have introduced a speed boundary set at 0.35 m/s, which separated those functionally better from those who recovered less.

In both, acute and chronic hemiplegic patients, we found considerable deviation from healthy pattern on the paretic leg, regardless of speed. Distribution of variance between principal components has moved, as mentioned above, towards the first principal component (first PC explaining up to 67% of total variance). Correlations between joint angles showed notably weaker correlation between hip and ankle, caused by a partial or complete loss of dorsiflexion movement. This has provoked stronger negative correlation of hip and knee.

The analysis of the nonparetic leg gave us an insight into rather unusual differences between acute and chronic hemiplegic patients. While all acute patients had a healthy-like pattern on the nonparetic leg, chronic patients formed two distinct groups. This partition was in accordance with previously set gait speed boundary. Those functionally better show either a healthy pattern on both legs or similar patterns as in acute patients. On the other hand, a chronic group that has not recovered a lot of their functionality shows modified patterns on both, the nonparetic and the paretic leg. If we look at the correlations, the nonparetic leg is characterized with negatively correlated hip and ankle, as well as negatively correlated hip and knee. The paretic leg, depending on a particular subject, shows various kinds of disturbed behavior.

Although chronic subjects have been involved in at least one conventional treatment, if not some additional, our study showed that not all of them benefited from it. Results from subjects in acute group indicate that in this early phase of therapy, all patients have more or less-disturbed pattern in the paretic leg and a healthy-like pattern on the nonparetic leg. If we look at the differences in the gait pattern of chronic group patients, we could conclude that those who recovered more, kept their healthy patterns on the nonparetic leg, and regained a healthy pattern on the paretic leg, or at least improved this pattern to some point. In subjects who made no particular progress in quality of gait, despite the fact that they were involved in rehabilitation for longer periods of time, we found disturbed gait patterns on both legs. This indicates that compensatory mechanisms evolved during rehabilitation process and not only affected the paretic leg, but also violated the existing healthy pattern of the nonparetic leg.

The most likely reason for the abnormal synergies is related to the task that was dominating the recovery of gait: faster gait and ability to cover longer distances. The obvious fact is that in both groups there was basically little “returning” to the normal synergies (Table 2).

The main finding from this study is that it is important to use kinematical data and apply stochastic analysis in order to provide adequate elements for analyzing the recovery that follows a treatment. We suggest that the use of the PCA characterizes better the specific features and abnormalities of the joint angles compared to some conventional techniques where only few nonconsecutive steps were used for the analysis [22–25]. This is especially in case of studying both acute and chronic stroke patients. While there are many studies about benefits of rehabilitation of patients in the acute recovery phase, there are still many doubts about success in chronic patients, who may require different techniques and approaches. Their treatment could benefit from better understanding of underlying mechanisms that PCA provides.

## Figures and Tables

**Table 1:** Basic characteristics of patients participating in the study.

	Chronic group (CG) 16 subjects		Acute group (AG) 11 subjects	
	Mean ± SD	Min/Max	Mean ± SD	Min/Max
Age (years)	63.2 ± 6.9	44/66	65.5 ± 7.8	42/69
Time after stroke (months)	39.9 ± 27.9	12/84	3 ± 1.7	1/6
Fugl-Meyer (FM) Score	22.50 ± 4.5	15/30	21.67 ± 5.33	12/30
Berg Balance (BB) score	36.17 ± 4.83	28/44	31.83 ± 6.22	21/41
Functional Ambulation Category (FAC) score	2.28 ± 0.78	2/3	2.05 ± 0.56	1/3
Height (cm)	171.3 ± 5.4	165/179	168 ± 7.1	159/176
Weight (kg)	68.1 ± 12.0	64/82	71.3 ± 12.5	61/80
Sex (female/male)	7/9		4/7	
Affected side (left/right)	10/6		8/3	

**Table 2:** The values of PCA for the healthy subjects and hemiplegic individuals divided into four groups based on the velocity of gait. Bold numbers show the category with significant difference (*P* < 0.05). Examples of “good” and “bad” recovery are shown in Figure 6.

GROUP		F1 + F2	F1	F2	
HEALTHY		88.3 ± 2.7	58.2 ± 2.7	29.2 ± 2.0	
*v* > 0.35 m/s	CG	86.82 ± 4.1	59.38 ± 3.8	35.31 ± 4.7	Non paretic leg
	AG	86.46 ± 5.0	57.99 ± 3.7	32.03 ± 4.9	
*v* < 0.35 m/s	CG	94.01 ± 4.9*	63.88 ± 3.8*	38.49 ± 5.3*	
	AG	89.01 ± 3.9	56.06 ± 6.0	30.95 ± 5.0	

*v* > 0.35 m/s	CG	92.99 ± 4.2*	67.04 ± 3.6*	19.97 ± 3.2*	Paretic leg
	AG	95.77 ± 4.4*	66.31 ± 5.2*	29.46 ± 3.5*	
*v* < 0.35 m/s	CG	98.15 ± 1.3*	64.89 ± 5.1*	20.96 ± 3.4*	
	AG	91.93 ± 3.6*	56.55 ± 4.4*	35.39 ± 4.1*	
